# Global prevalence of Rett syndrome: systematic review and meta-analysis

**DOI:** 10.1186/s13643-023-02169-6

**Published:** 2023-01-16

**Authors:** Uarda Petriti, Daniel C. Dudman, Emil Scosyrev, Sandra Lopez-Leon

**Affiliations:** 1 Cognizant Solution, Amsterdam, the Netherlands; 2 Former Rutgers University Ernest Mario School of Pharmacy, Piscataway, NJ USA; 3 Former Quantitative Safety & Epidemiology, Novartis Pharmaceuticals Corporation, East Hanover, NJ USA; 4 Quantitative Safety & Epidemiology, Novartis Pharmaceuticals Corporation, One Health Plaza, Building 339-1131, East Hanover, NJ 07936-1080 USA; 5 Rutgers Center for Pharmacoepidemiology and Treatment Science, Institute for Health, New Brunswick, NJ USA

**Keywords:** Epidemiology, Incidence, *MECP2*, Meta-analysis, Neurodevelopmental disorders, Prevalence, Rare diseases, Rett syndrome (RTT), Systematic literature review

## Abstract

**Background:**

Rett syndrome is a rare, severe neurodevelopmental disorder. Almost all cases occur in girls, in association with spontaneous (non-inherited) mutations involving the *methyl-CpG-binding protein 2* gene located on the X chromosome. Diagnostic criteria for typical Rett syndrome require a period of regression, followed by recovery or stabilization, and fulfillment of all four main criteria (loss of purposeful hand skills, loss of spoken language, gait abnormalities, and stereotypic hand movements). Our objective was to estimate the prevalence of Rett syndrome in the general population, stratified by sex.

**Methods:**

We conducted a search of PubMed, Embase, Web of Science, Cochrane Library, LILACS, and LIVIVO to retrieve studies published in English between Jan. 1, 2000, and June 30, 2021. Pooled prevalence with a 95% confidence interval (CI) was estimated using a random-effects meta-analysis based on a generalized linear mixed model with a logit link.

**Results:**

Ten eligible studies were identified (all in females), with a combined sample size of 9.57 million women and 673 Rett syndrome cases. The pooled prevalence estimate (random effects) was 7.1 per 100,000 females (95% CI: 4.8, 10.5, heterogeneity *p* < 0.001). Despite greatly variable precision of estimation, all estimates were compatible with a prevalence range of approximately 5 to 10 cases per 100,000 females based on their respective 95% CIs.

**Conclusion:**

These findings may facilitate planning of therapeutic trials in this indication in terms of target sample size and accrual times.

**Supplementary Information:**

The online version contains supplementary material available at 10.1186/s13643-023-02169-6.

## Background

Rett syndrome (RTT) is a rare, severe neurodevelopmental disorder that affects almost exclusively females. The syndrome was first described in 1966 by Andreas Rett in the German medical literature [1]. However, RTT was not internationally recognized until 1983 when Hagberg et al. described the first cases in the English language literature [2]. In 1999, Amir et al. [3] identified mutations on the *methyl-CpG binding protein 2* (*MECP2*) gene, which is located on the Xq28 chromosome band and encodes *MECP2* in RTT patients. Mutations in *MECP2* have been detected in approximately 95–97% of typical RTT cases and 85% of atypical RTT [1]. In addition to patients with RTT, mutations have been identified in individuals who do not have the clinical features of RTT. Because *MECP2* mutations are neither necessary nor sufficient to make the diagnosis of RTT, the disorder remains a clinical diagnosis [4].

Development appears normal during the first 6 to 18 months of life but is followed by regression of motor and language skills. The clinical phenotype of RTT is broad and can be classified into two main categories: typical (classic) RTT and atypical (variant) RTT. Diagnostic criteria for typical RTT require a period of regression, followed by recovery or stabilization, and fulfillment of all four main criteria (loss of purposeful hand skills, loss of spoken language, gait abnormalities, and stereotypic hand movements) [4]. In some cases, deceleration of head growth can be one of the first signs of RTT. Further manifestations can include seizures, autistic features, intermittent breathing abnormalities, autonomic nervous system dysfunction, cardiac abnormalities, and sleep disturbances. Atypical RTT encompasses variants of RTT that have many but not all of the clinical features of typical RTT. The three defined atypical RTT variants are preserved speech, early onset seizure, and congenital variants [2].

Kirby et al. examined the longevity of patients with RTT in a cohort study conducted in the USA and Canada (*N* = 1928 subjects) [5] and found that most RTT-related deaths occurred before the age of 25 years. The researchers reported an overall survival of approximately 78% at age 25 years.

No cure nor effective disease-modifying therapy currently exists for RTT. Several pharmacologic treatments, including glatiramer acetate, dextromethorphan, and trofinetide, have been investigated in small clinical trials. Modest benefits were reported for endpoints such as gait velocity, respiratory function, seizures, and certain cognitive and behavioral parameters [6–8]. Gene therapy, which is in the drug development phase, demonstrates promise [9, 10].

One of the rate-limiting factors in the development of new pharmacologic therapies for RTT is the low prevalence of the disease, which makes conducting large clinical trials for this indication difficult. To date, no meta-analyses have reported on the prevalence of RTT. One meta-analysis that focused on the prevalence of autism spectrum disorders (ASDs) reported that 61% of children with RTT have ASD [11]. The aim of this systematic review and meta-analysis is to review the current literature pertaining to RTT and to estimate the prevalence of RTT in the general population, stratified by sex. These results may facilitate planning of future clinical trials for this indication in terms of target sample size and accrual times.

## Methods

### Search strategy

We performed a systematic search of electronic databases (PubMed, Embase, Web of Science, Cochrane Library, LILACS, and LIVIVO). Search strategies combined relevant terms for the disease (Rett, MECP2) with those for the occurrence (prevalence, incidence, epidemiology) (see Supplement 1: Database Search Strategies). The search was limited to records published from 1 January 2000 to 30 June 2021. We established the date limit of 2000 for study inclusion because the association of RTT with the MECP2 mutation was recognized in the year 1999 [3]. While the MECP2 mutation is neither necessary nor sufficient for the diagnosis of RTT, the current diagnostic criteria [4] acknowledge that identification of this mutation may result in a diagnosis of “possible RTT,” which can be further revised to a definite RTT diagnosis when the clinical criteria are fulfilled. The search was limited to publications in the English language and to human patients, and no geographic restriction was applied.

### Study selection

Original, peer-reviewed articles reporting the prevalence and/or incidence of RTT (or sufficient data to calculate them) in the general population within a defined geographical area were eligible for inclusion. Review articles, conference abstracts, or unpublished manuscripts were excluded. If there were studies reporting duplicate data, the study with the most up-to-date and complete data was included. The full inclusion and exclusion criteria are listed in Table 1.

**Table 1:** Study inclusion and exclusion criteria

Category	Inclusion criteria	Exclusion criteria
Study design/publication type	-Observational studies (prospective, retrospective, cross-sectional, surveillance studies) -Meta-analysis or systematic reviews (to check the reference lists) -Human studies	-Randomized controlled trials (RCTs) -Narrative review -Conference abstracts, unpublished manuscripts, expert opinions, editorials, comments, letters to the editor -Animal studies -Genetic or molecular laboratory studies
Country	All countries/worldwide	No countries excluded
Study topic/subject	Diagnosed RTT syndrome	Other or unspecified conditions
Study population	General population (also stratified by, e.g., gender, age, sex, geographical area, etc.)	Other populations
Outcomes of interest	Prevalence/incidence/proportion of diagnosed RTT	Other outcomes

*RCTs* Randomized controlled trials, *RTT* Rett syndrome

Two reviewers (UP and DCD) examined the titles and abstracts of the retrieved publications in duplicate, and the full texts of selected articles were subsequently screened in duplicate. Reference lists of the included articles as well as of the review articles were manually screened to check for additional relevant articles. All records were transferred into the EndNote reference manager, where duplicates were automatically removed. In both screening steps, we achieved concordance of more than 98%. Disagreements on eligibility were resolved by discussion with a third reviewer (SLL). The Preferred Reporting Items for Systematic Reviews and Meta-Analysis standards 2021 guidelines were followed (Supplemental Table 2) [12]. The study protocol was not preregistered.

**Table 2:** Characteristics of the included studies

**Authors, year**	**Country**	**Study period**	**Study design/population**	**Age of study population, years**	**Diagnosis criteria for Rett syndrome**	**Cases**
Bienvenu et al. [13] (2006)	France	Born 1989–2000	National population-based: Rett registry	4–15	Clinical and genetic	Rett
Magnússon et al. [14] (2001)	Iceland	Born 1974–1993	National population-based: registers of tertiary hospitals	5–24	Clinical, pediatrician, psychiatrist (ICD-10)	ASD with Rett
Strømme et al. [15] (2000)	Norway, Akershus	Born 1980–1985	Population-based: multiple search strategies	NR	Clinical (ICD-10 [code F84.2])	MR with Rett
Isaksen et al. [16] (2012)	Norway, Oppland, and Hedmark	Born 1996–2002	Population-based: multiple sources like registries, schools, hospitals, and public health services	6–12	Clinical, pediatrician, neurologists (ICD-10 [code F84.2])	ASD with Rett
Sarajlija et al. [17] (2015)	Serbia	1981–2001	National population-based: registers of Mother and Child Health Institute of Serbia	<18	Clinical and genetic	Rett
Aguilera et al. [18] (2007)	Spain, Seville	2002–2003	Population-based: all schools in the area	3–21	Clinical, confirmed by schools (DSM-IV, ICD-10)	ASD with Rett
Fombonne et al. [19] (2003)	UK, England, Wales, Scotland	1999	Population-based: Sample of Child Benefit Register	5–15	Clinical, psychiatrist (DSM-IV, ICD-10)	PDD with Rett
Chakrabarti et al. [20] (2005)	UK, Midlands	Born 1996–1998	Population-based: Child Health Surveillance	4–6	Clinical, psychiatrist (DSM-IV)	PDD with Rett
Fehr et al. [21] (2011)	Australia	Born 1976–2006	National population-based: multiple sources	5–32	Clinical and genetic	Rett
Wong et al. [22] (2007)	China, Hong Kong West	2006	Population-based: tertiary hospital	<35	Clinical and genetic (DSM-IV)	Rett

*ASD* Autism spectrum disorders, *DSM-IV* Diagnostic and Statistical Manual of Mental Disorders, Fourth Edition, *ICD-10* International Classification of Diseases, Tenth Revision, *MR* Mental retardation, *NR* Not reported, *PDD* Pervasive developmental disorder, *UK* United Kingdom

### Data extraction and quality assessment

Relevant data were extracted using a standardized data collection form and included information on study design, study population, data collection period, location, diagnostic criteria/definitions for RTT, and sources of case ascertainment (Table 2). Prevalence estimates of RTT or raw numbers were recorded. The quality of eligible studies was assessed using the MetaXL User Guide Version 5.3 [23] predefined criteria list and included population representativeness, catchment area, disease assessment, and statistical methods. A quality score, which ranged from 0 to 11, was estimated, with a greater score indicating a better study quality (Table 3).

**Table 3:** MetaXL quality assessment score

**Study**	**1. Population and observation period**	**2. Diagnostic criteria**	**3. Methods of case ascertainment**	**4. Administration of measurement protocol**	**5. Catchment area**	**6. Prevalence measure**	**Total (max: 11)**
Aguilera et al. [18] (2007)	1	1	2	3	2	2	11
Bienvenu et al. [13] (2006)	1	1	2	1	2	2	9
Isaksen et al. [22] (2012)	1	1	2	3	2	2	11
Chakrabarti et al. [20] (2005)	1	1	2	2	2	1	9
Fombonne et al. [19] (2003)	1	1	1	3	2	2	10
Magnússon et al. [14] (2001)	1	1	1	1	2	2	8
Strømme et al. [15] (2000)	1	1	2	3	2	2	11
Sarajlija et al. [17] (2015)	1	1	1	2	2	2	9
Fehr et al. [21] (2011)	1	1	2	2	2	2	10
Wong et al. [22] (2007)	1	1	2	2	2	2	10

1) Yes = 1, no = 0

2) Diagnostic system reported = 1. Own system/symptoms described/no system/not specified = 0

3) Community survey/multiple institutions = 2. Inpatient/inpatients and outpatients/case registers = 1. Not specified = 0

4) Administered interview = 3. Systematic case note review = 2. Chart diagnosis/case records = 1. Not specified = 0

5) Broadly representative (national or multi-site survey) = 2. Small area/not representative (single community, single university) = 1. Convenience sampling/other (primary care sample/treatment group) = 0

6) Point prevalence (e.g., 1-month prevalence = 2; 12-month prevalence = 1; lifetime prevalence = 0)

### Data synthesis and meta-analysis

All studies that were included used a cross-sectional design and estimated the prevalence, which was defined as the number of existing RTT cases expressed relative to the population size, in a well-defined population at one specific point in time. Studies were included in the meta-analysis if they reported the number of cases and the sample denominator or sufficient information to calculate the prevalence. The random-effects estimate of the pooled prevalence with a 95% confidence interval (CI) was calculated based on the generalized linear mixed model (GLMM) with a logit link function [24]. This approach results in valid inference with common or rare outcomes [24]. A heterogeneity test *p*-value for the null hypothesis of equal study-specific prevalence parameters was derived from the GLMM model [24]. The *I*^2^ statistic was calculated as *I*^2^ = (*Q* − df)/*Q*, where *Q* = CINV (1 − *p*-value, df), CINV is the chi-square inverse, and df = (number of studies − 1) [25]. For a visual examination of heterogeneity, individual study-specific prevalence estimates with 95% CIs were displayed together with the pooled prevalence estimate in a forest plot [25]. The 95% CIs for the study-specific prevalence parameters were calculated based on the exact binomial method [26]. The pooled prevalence estimate was based on the random-effects model due to evidence of heterogeneity, and “a priori” low plausibility of the homogeneity hypothesis, considering that the prevalence of most medical conditions is known to vary geographically and over time. A funnel plot of the estimated prevalence versus the margin of error (half-length of the 95% CI) was constructed to examine the variability of the study-specific estimates as a function of their estimated precision. In the absence of substantial heterogeneity, more precise estimates (i.e., those with the smaller margin of error) are expected to have relatively little spread in the plot, while outliers, if present, are expected to have large error margins. Outliers with small error margins are evidence of heterogeneity. Unlike studies of the treatment effects, however, prevalence studies are neither “positive” nor “negative.” Hence, the funnel plot does not provide information on publication bias in prevalence studies. Similarly, while power analysis is sometimes recommended for meta-analyses of treatment effects, where the absence of the treatment effect constitutes a natural null hypothesis [27, 28], this is usually not applicable to prevalence studies, where the focus is on point and interval estimation of the average prevalence parameter, as in the present work. Meta-analysis was performed based on all eligible studies combined and by subgroups defined by the use of genetic testing in the studies. All analyses were performed in SAS 9.4. The analysis code is available as Supplement 3.

## Results

### Study selection and characteristics

**Fig. 1:**
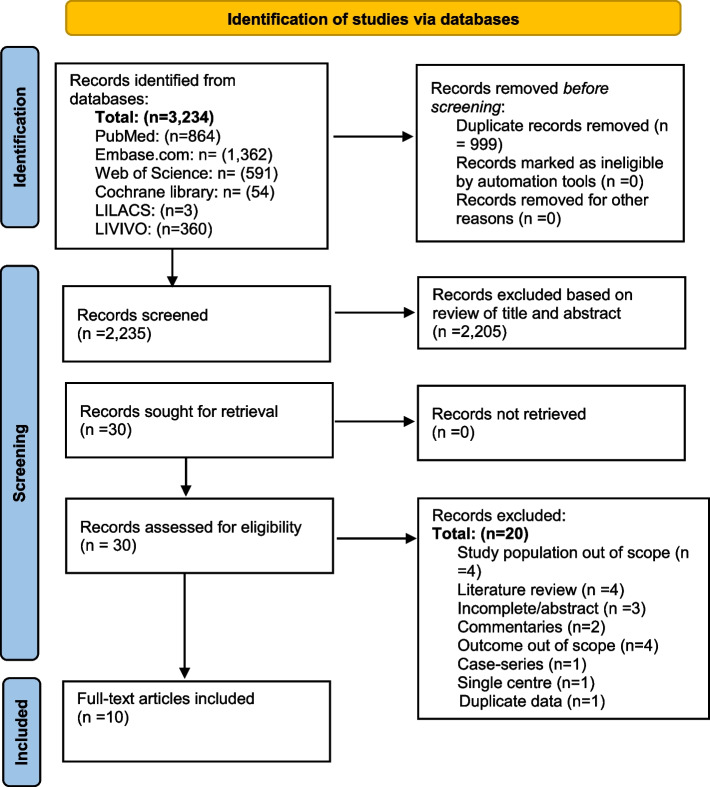
PRISMA 2020 flow diagram for new systematic reviews. Searches of databases and registers only were included. PRISMA, Preferred Reporting Items for Systematic Reviews and Meta-Analyses

A total of 3234 articles were identified. After reviewing the titles and abstracts, 30 articles were selected for full-text evaluation. A review of the references of these studies identified one other article for inclusion. The review of the 30 full-text articles led to the selection of 10 studies that were considered relevant for the present review. These 10 studies were considered of sufficient quality, according to the MetaXL guidelines on assessing the study quality, and all 10 studies were included in the meta-analysis. A summary of the study quality assessment score is presented in Table 3. A summary of the article selection process is presented in Fig. 1.

Four studies had an objective of estimating the prevalence of RTT: Bienvenu et al. from France, Sarajlija et al. from Serbia, Wong et al. from China, and Fehr et al. from Australia [13, 17, 21, 22]. Six studies had a broader diagnosis surveyed but presented stratifications of which RTT was a category [14–16, 18–20]. In our analyses, only the RTT information was included.

The sizes of the study populations ranged from 5227 to 4,337,627 and the majority of studies (five) included girls younger than age 18 years. Three of the studies included patients for which the age of the study population was 3 to 21 years [18], 5 to 24 years [14], or 5 to 32 years [21]. One other study observed patients younger than 35 years [22], while the age of the study population was not reported in one study [15]. All studies were population-based, and four were nationwide in their respective countries [13, 14, 17, 21]. Sampling methods differed between studies. For example, some studies reported multiple data sources (registers, schools, hospitals, and public health services) to ascertain RTT cases, whereas other studies used surveillance data or registers of tertiary hospitals. Table 2 provides characteristics of the included studies.

### Meta-analysis

**Fig. 2:**
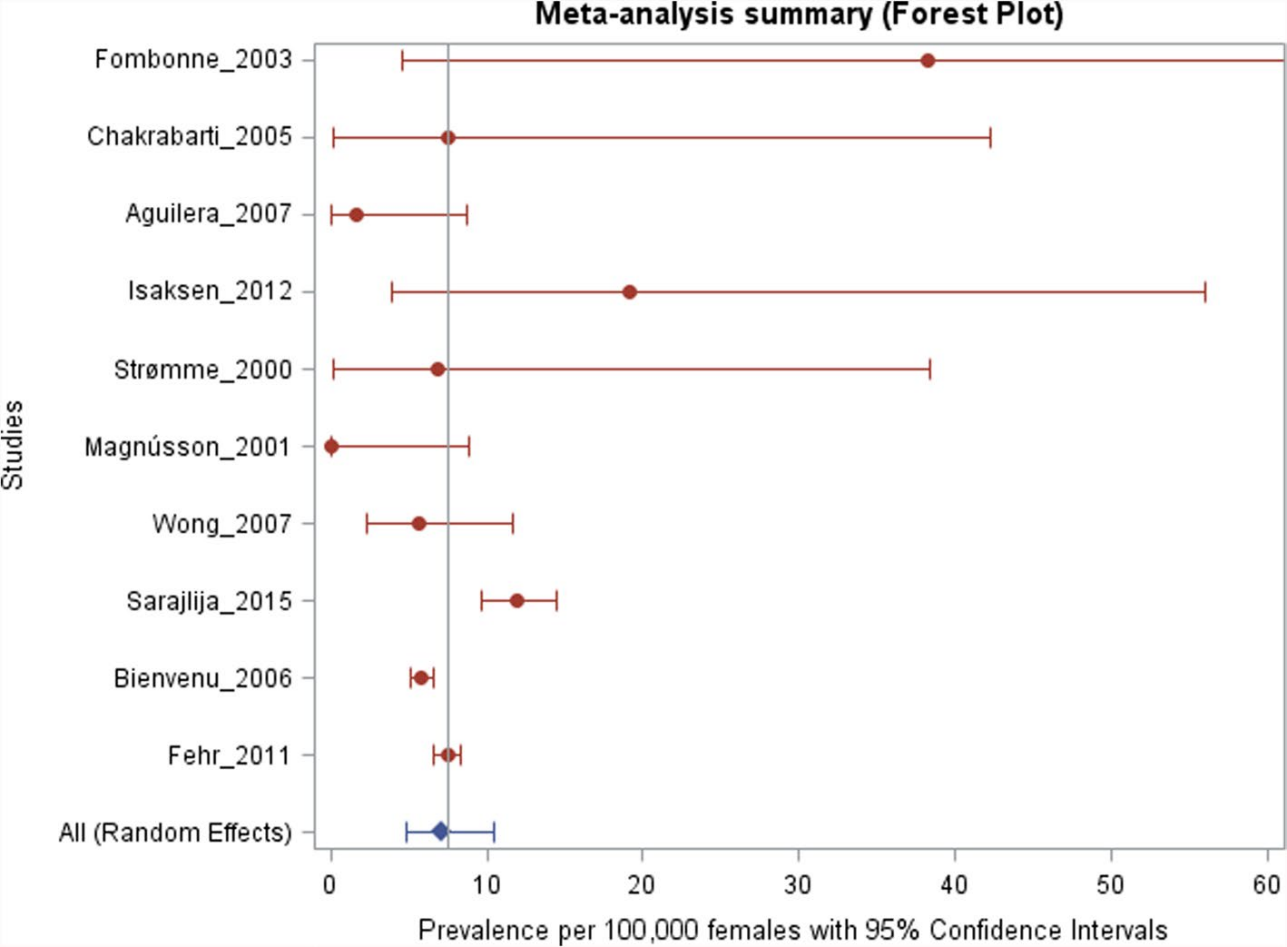
Forest plot demonstrating the study-specific prevalence of Rett syndrome estimates per 100,000 females (95% confidence interval)

**Fig. 3:**
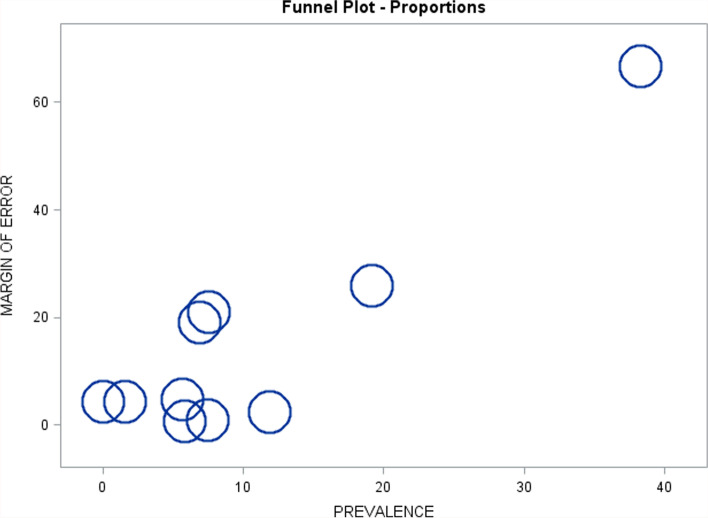
Funnel plot demonstrating the estimated prevalence of Rett syndrome compared with margin of error (half-length of the 95% confidence interval) to examine the variability of the study-specific estimates as a function of their estimated precision

The meta-analysis is presented in Table 4, with forest and funnel plots presented in Figs. 2 and 3, respectively. Study-specific prevalence estimates per 100,000 females (95% CI) ranged from 0.0 (0.0, 8.8) to 38.3 (4.6, 138.0), with a highly significant heterogeneity test (*p* < 0.001, *I*^2^ = 0.831). However, much of this variability was due to a few imprecise estimates, such as Isaksen et al. [16], Fombonne et al. [19], Chakrabarti et al. [20], and Strømme et al. [15]. More precise prevalence estimates such as those reported by Fehr et al. [21], Bienvenu et al. [13], and Sarajlija et al. [17] were not highly variable (as observed in Figs. 2 and 3), and although some had non-overlapping 95% CIs (such that statistical evidence against the null hypothesis of equal prevalence parameters was rather strong), the magnitude of this variability was not great. The pooled prevalence estimate based on all eligible studies (random-effects model) was 7.1 cases per 100,000 females (95% CI: 4.8, 10.5). Pooled prevalence estimates within the two subgroups defined by the use of genetic testing in the studies were of similar magnitude and not significantly different from each other (*p* = 0.84), although the estimate in the first subgroup was much less precise than in the second due to the differences between the subgroups in the sample sizes and the case counts (Table 4). Interestingly, most estimates from the European region were of similar orders of magnitude as those from China and Australia. Despite greatly variable precision of estimation, all estimates in Table 4 are compatible with a prevalence range of approximately 5 to 10 cases per 100,000 females based on their respective 95% CIs. All studies had a quality score of eight points or greater.

**Table 4:** Meta-analysis of Rett prevalence (per 100,000 females)

***Case definition criteria*** **Study**	**Country**	**Rett** **cases**	**Female** **population**	**Prevalence** **estimate**	**95%** **LCL**	**95%** **UCL**
*Clinical diagnosis only*^a^						
Fombonne et al. (2003) [19]	UK	2	5227	38.3	4.6	138.0
Chakrabarti et al. (2005) [20]	UK	1	13202	7.6	0.2	42.2
Aguilera et al. (2007) [18]	Spain	1	63675	1.6	0.0	8.7
Isaksen et al. (2012) [16]	Norway	3	15662	19.2	4.0	56.0
Strømme et al. (2000) [15]	Norway	1	14542	6.9	0.2	38.3
Magnússon et al. (2001) [14]	Iceland	0	41896	0.0	0.0	8.8
Pooled prevalence		8	154204	6.7	2.0	22.0
*Clinical diagnosis + genetic testing*^b^						
Wong et al. (2007) [22]	China	7	123968	5.6	2.3	11.6
Sarajlija et al. (2015) [17]	Serbia	102	857142	11.9	9.7	14.4
Bienvenu et al. (2006) [13]	France	251	4337627	5.8	5.1	6.5
Fehr et al. (2011) [21]	Australia	305	4094386	7.4	6.6	8.3
Pooled prevalence		665	9413123	7.6	5.4	10.8
*All studies*^c^		673	9567327	7.1	4.8	10.5
Pooled prevalence						

^a^Genetic testing not reported in the study publications (*n* = 6 studies, *Q* = 13.0; df = 5; heterogeneity *p* = 0.0231; *I*^2^ =0.616)

^b^Genetic testing reported in the study publications (*n* = 4 studies, *Q* = 36.6; df = 3; heterogeneity *p*<0.001; *I*^2^ = 0.918)

^c^All eligible publications (*n* = 10 studies, *Q* = 53.3; df = 9; heterogeneity *p*<0.001; *I*^2^ = 0.831, subgroup difference *p*=0.84)

*LCL* Lower confidence limit, *UCL* Upper confidence limit

## Discussion

This is the first systematic review and meta-analysis of RTT that reports pooled prevalence of RTT in the general female population. Our pooled prevalence estimate of 7.1 per 100,000 females (95% CI: 4.8, 10.5) is in line with the estimate reported on Orphanet (http://orpha.net; 10 per 100,000 live female births) [29], though the Orphanet estimate is limited by lack of a published description of its methods and data sources.

No studies that included patients with RTT older than 35 years were included. Studies report that, after reaching 25 years of age, adults with RTT have a mortality rate similar to the general population [5]. If that is indeed the case, the pooled prevalence estimates presented could be extrapolated to the general population. However, future studies should include patients of all ages to determine if the prevalence changes with age.

The strengths of this study were that the results encompassed the prevalence estimates from several nations and covered many different patient populations. Similar estimates were obtained for many different populations, and the true prevalence of RTT did not vary substantially from one region to another. To supplement the clinical criteria, some studies also used the *Diagnostic and Statistical Manual of Mental Disorders, Fourth Edition*, *International Classification of Disease, Tenth Revision*, and genetic criteria.

The present meta-analysis has several limitations. The majority of the studies involved only females younger than age 24 years. No studies assessing the prevalence of all age groups have been completed. Subgroup information was not available from the original publications, so meta-analysis by subgroups could not be performed. Concerning the diagnostic criteria, the studies could have used different criteria given that these have changed in 1985, 2002, and 2010. Surprisingly, no studies from the USA were published during the review period (1 January 2000 to 30 June 2021). One study was identified, published in 1993 from a large population-based registry in Texas, in which the prevalence of classic RTT was estimated to be 4.4 per 100,000 females [30]. This is in line with what has been reported in the studies presented here (5 to 10 cases per 100,000 females). The protocol for this systematic review and meta-analysis was not preregistered on Prospective Register of Systematic Reviews (PROSPERO) or elsewhere, which is acknowledged as a limitation.

## Conclusions

In summary, this is the first meta-analysis that estimates the prevalence of RTT. The results suggest that the prevalence remained stable for the last 20 years in the range of 5 to 10 cases per 100,000 females, without substantial regional variability. These findings may facilitate planning of therapeutic trials in this disease, especially for target sample size and accrual times.

## Supplementary Information

**Additional file 1: Supplement 1.** Database search strategies.

**Additional file 2: Supplemental Table 2.** PRISMA checklist.

**Additional file 3.** SAS code.

## Data Availability

Not applicable.

## Abbreviations

ASD: Autism spectrum disorder
CI: Confidence interval
GLMM: Generalized linear mixed model
*MECP2*: *Methyl-CpG binding protein 2* gene
PROSPERO: Prospective Register of Systematic Reviews
RTT: Rett syndrome
SAS: Statistical Analysis Systems
