# Supplementary material for: Global prevalence of Rett syndrome: systematic review and meta-analysis
Source: Syst Rev. 2023 Jan 16;12:5. doi: 10.1186/s13643-023-02169-6 (PMC9841621; doi:10.1186/s13643-023-02169-6)
Supplement: Supplementary file 1 — Additional file 1: Supplement 1. Database search strategies. [file 13643_2023_2169_MOESM1_ESM.docx]

**Supplement 1: Database search strategies**

**PubMed Search:**

**("Rett*" OR "Rett syndrome" OR "MECP2") AND ("prevalence" OR "incidence" OR "epidemiology")**

Date of the search: 30/06/2021

Langue limits: English

Date limits: from 2000-2021

Filters: Human subjects

Number of hits: 864

**Emabse.com Search:**

**('rett*' OR 'rett syndrome' OR 'mecp2') AND ('prevalence' OR 'incidence' OR 'epidemiology')**

Date of the search: 30/06/2021

Langue limits: English

Date limits: from 2000-2021

Filters: Human subjects

Number of hits: 1,362

**Web of Science Search:**

**TS= (("Rett*" OR "Rett syndrome" OR "MECP2") AND ("prevalence" OR "incidence" OR "epidemiology"))**

Date of the search: 30/06/2021

Langue limits: English

Date limits: from 2000-2021

Filters: Human subjects

Number of hits: 591

**Cochrane library Search:**

**("Rett*" OR "Rett syndrome" OR "MECP2") AND ("prevalence" OR "incidence" OR "epidemiology")**

Date of the search: 19-07-2022

Date limits: 2000-2021

Number of hits: 54

**LILACS Search:**

**("Rett*" OR "Rett syndrome" OR "MECP2") AND ("prevalence" OR "incidence" OR "epidemiology")**

Date of the search: 19-07-2022

Filters: English language

Number of hits: 3

**LIVIVO Search:**

**("Rett*" OR "Rett syndrome" OR "MECP2") AND ("prevalence" OR "incidence" OR "epidemiology")**

Date of the search: 19-07-2022

Filters: English language, Article (document type), 2000-2001 (years of publication)

Number of hits: 360
